# Supplementary material for: An Improved Reference Gene for Detection of “Candidatus Liberibacter asiaticus” Associated with Citrus Huanglongbing by qPCR and Digital Droplet PCR Assays
Source: Plants (Basel). 2021 Oct 5;10(10):2111. doi: 10.3390/plants10102111 (PMC8540500; doi:10.3390/plants10102111)
Supplement: Supplementary file 1 [file plants-10-02111-s001.zip › plants-1398554-supplementary.pdf]

Supplementary information:

CAAAGGTAGTAGAAGGCTTGATTGAGATTCTTTGGCGACCAAAAAGTTTATCTGCTAGTCTCTGTATTCATTTCTTA
TTTTGCATGCAG**GGACTGATAGTCAGCTCCCGGAAAGATTCACTTCAACACTTCAAGAAGCCTTGGGCTCATGAACA**
TTGCATGCAGGGACTGATAG *ACACTTCAAGAAGCCTTGGGCTCA GAACA*

MDH_F MDH_P

TGAGCCTGTCAACAATCTCTTAGATGCTGTCAAGGTGTTACAGACGTTTCTTTGATTAATGTTCAGTTTTTTCAGTG
TGAGCCTGTCAACAATC
MDH_R
TTTGTGGGTCCTTAAACTTTTACTCTCTTTGTCATAAGGTA**ATCAAGCCAACAATTTTGATTGGATCATCTGGAGTT**
GGAAGAACTTTTACAAAAGAAGTAATCGAGGCCATGGCCTCCTTCAATGAGGTAGTGTTTCAGGCTCTCCTTTGGCT
TATAAGGAAATTTAATTTTTGTTCTCTTTTCAAACGTATGTTTTAAGTGGGAGAAATTGAGCCTAAAGTGGTTAATG
CTGGCAG**AAACCTCTCATCCTGGCTCTCTCCAACCCAACCTCACAGTCAGAATGTACAGCTGAAGAAGCTTACACTT**
GGAGTAAGGTACAAACAATAATTCTGCAACACTTTCCAGGCTTCAACTAAGTTGGGGATGCTATTTTGTTAAGTAGA
CCTGTATTTTTCTACACACAGAAAAAACTAACTATTGTTATTTACGTTCCTTCTGCTTTTGATTTGAAG**GGTCGTGC**
AATTTTCGCTAGTGGAAGTCCATTTGATCCTTTTGAATACAATGGAAAAGTTTTTGTTCCTGGCCAGGTCTATAATC
ATAATCATTTTGCATTTGCTAAGATTCCTATAATCATATTTCTGTTATATTTTTTAATAATTACTAGTTATTGAATA
GTAGCTGAACTTAGAACGCTTGACATTTTTGCTTTTCCCGTGAATCCAGGCCAACAATGC

Figure S1. Sequence of a 906 bp region of citrus malate dehydrogenase used for designing primers and probe for qPCR and ddPCR assays. The exons in the region are indicated in bold, underlined, highlighted text. The primers and probe sequences are shown in italics. Note that the 5' ten bases of the forward primer are in the intron.

Table S1. A list of selected taxa of Aurantioideae used for sequencing 900 bp region of the malate dehydrogenase gene. Accession numbers refer to sequences deposited in Genbank. Taxa with two accession numbers indicate the presence of two haplotypes. Three non-aurantioid taxa were included in the analysis.

No.	Genus	species	Cultivar	Subfamily	Subtribe	Accession
1	*Aegle*	*marmelos*	Indian Bael fruit	Aurantioideae	Balsamocitrinae	JN612891.1
2	*Aeglopsis*	*chevalieri*	Chevalier's Aeglopsis	Aurantioideae	Balsamocitrinae	JN612892.1
3	*Afraegle*	*paniculata*	Nigerian powder flask fruit	Aurantioideae	Balsamocitrinae	JN676196.1
4	*Atalantia*	*ceylanica*	Ceylon Atalantia	Aurantioideae	Citrinae	EU254083.1
5	*Atalantia*	*citroides*	Cochin china Atalantia	Aurantioideae	Citrinae	JN612893.1
6	*Atalantia*	*monophylla*	Indian Atalantia	Aurantioideae	Citrinae	JN612894.1
7	*Balsamocitrus*	*daweii*	Uganda powder flask fruit	Aurantioideae	Balsamocitrinae	JN612895.1
8	*Bergera*	*koenigii*	Curry leaf	Aurantioideae	Clauseninae	JN612897.1
9	*Burkillanthus*	*malaccensis*	Malay ghost-lime	Aurantioideae	Citrinae	JN612898.1
10	*Choisya*	*ternata*		Rutoideae	Zanthoxyleae	JN676195.1
11	*Citropsis*	*daweana*	Mozambique cherry-orange	Aurantioideae	Citrinae	JN612899.1
12	*Citropsis*	*gabunensis*	Gabon cherry-orange	Aurantioideae	Citrinae	EU254084.1

13	*Citropsis*	*schweinfurthii*	Uganda cherry-orange	Aurantioideae	Citrinae	JN612900.1
14	*Citrus*	*amblycarpa*	Nasnaran mandarin	Aurantioideae	Citrinae	EU254103.1; EU254104.1
15	*Citrus*	*aurantifolia*	Mexican lime	Aurantioideae	Citrinae	EU254096.1; EU254097.1
16	*Citrus*	*aurantium*	Rubidoux sour	Aurantioideae	Citrinae	EU254115.1; EU254116.1
17	*Citrus*	*halimi*	Mountain citron	Aurantioideae	Citrinae	KT175681.1; KT175682.1
18	*Citrus*	*hanaju*	Hanayu papeda	Aurantioideae	Citrinae	KT175649.1; KT175650.1
19	*Citrus*	*hongheensis*	Honghe papeda	Aurantioideae	Citrinae	EU254098.1; EU254099.1
20	*Citrus*	*ichangensis*	Ichang papeda	Aurantioideae	Citrinae	EU254094.1; EU254095.1
21	*Citrus*	*limettioides*	Palestine Sweetlime	Aurantioideae	Citrinae	KT175677.1; KT175678.1
22	*Citrus*	*longispina*	Winged lime	Aurantioideae	Citrinae	EU254119.1; EU254120.1
23	*Citrus*	*maxima*	Siamese sweet pummelo	Aurantioideae	Citrinae	EU254111.1; EU254112.1
24	*Citrus*	*medica*	Ninger giant citron	Aurantioideae	Citrinae	KT175679.1; KT175680.1
25	*Citrus*	*nippokoreana*	Korai mandarin	Aurantioideae	Citrinae	KT175675.1; KT175676.1
26	*Citrus*	*nobilis*	King tangor	Aurantioideae	Citrinae	EU254092.1; EU254093.1
27	*Citrus*	*reshni*	Cleopatra mandarin	Aurantioideae	Citrinae	JN612910.1; JN612909.1
28	*Citrus*	*reticulata*	Ponkan mandarin	Aurantioideae	Citrinae	EU254132.1; EU254133.1
29	*Citrus*	*sinensis*	Harts Tardiff Valencia	Aurantioideae	Citrinae	JN612919.1; JN612918.1
30	*Citrus*	*unshiu*	Frost Owari satsuma	Aurantioideae	Citrinae	EU254100.1
31	*Citrus*	*webberi*	Kalpi lime	Aurantioideae	Citrinae	EU254092.1
32	*Citrus*	*wintersii*		Aurantioideae	Citrinae	JN612905.1
33	*Clausena*	*excavata*	Pink wampee	Aurantioideae	Clauseninae	JN612906.1
34	*Clausena*	*harmandiana*		Aurantioideae	Clauseninae	JN612907.1
35	*Clymenia*	*polyandra*		Aurantioideae	Citrinae	JN612908.1
36	*Eremocitrus*	*glauca*	Australian desert lime	Aurantioideae	Citrinae	JN612903.1
37	*Flindersia*	*australis*	Australian teak	Flindersioideae		JN676194.1
38	*Glycosmis*	*mauritiana*		Aurantioideae	Clauseninae	JN612910.1
39	*Glycosmis*	*pentaphylla*	Orange berry/Gin berry	Aurantioideae	Clauseninae	JN612911.1

40	*Glycosmis*	*trichanthera*		Aurantioideae	Clauseninae	JN612912.1
41	*Merillia*	*caloxylon*		Aurantioideae	Merrilliinae	JN612913.1
42	*Microcitrus*	*australasica*	Australian finger lime	Aurantioideae	Citrinae	EU254132.1
43	*Micromelum*	*minutum*		Aurantioideae	Micromelinae	JN612914.1
44	*Monathocitrus*	*cornuta*	Spotseed-lime	Aurantioideae	Triphasiinae	JN612915.1
45	*Murraya*	*paniculata*	Orange jasmine	Aurantioideae	Clauseninae	JN612919.1
46	*Naringi*	*crenulata*	Hesperethusa	Aurantioideae	Citrinae	JN612920.1
47	*Oxanthera*	*neo-caledonica*	False orange	Aurantioideae	Triphasiinae	JN612921.1
48	*Pamburus*	*missionis*		Aurantioideae	Triphasiinae	JN612923.1
49	*Paramignya*	*lobata*		Aurantioideae	Triphasiinae	JN612924.1
50	*Paramignya*	*scandens*		Aurantioideae	Triphasiinae	JN612925.1
51	*Pleiospermium*	*latialatum*	North Borneo orangeaster	Aurantioideae	Citrinae	JN612926.1
52	*Poncirus*	*trifoliata*	Pomeroy	Aurantioideae	Citrinae	EU254129.1
53	*Ruta*	*graveolens*	Rue	Rutoideae		JN612931.1
54	*Severinia*	*buxifolia*	Chinese box orange	Aurantioideae	Citrinae	JN612927.1
55	*Swinglea*	*glutinosa*	Tabog	Aurantioideae	Balsamocitrinae	JN612928.1
56	*Triphasia*	*trifolia*	Trifoliate limeberry	Aurantioideae	Triphasiinae	JN612929.1
57	*Wenzelia*	*dolichophylla*		Aurantioideae	Triphasiinae	JN612930.1

Table S2. Analysis of citrus leaf samples by qPCR and digital droplet PCR. Freeze-dried leaf samples were used for singleplex ddPCR (for detecting CLas) and by duplex qPCR (for COX and CLas) assays. Out of a total of 440 samples tested, 57 were positive by both assays. Results from 32 representative samples where the ddPCR reaction contained less than 50% positive droplets are shown in the table. Samples with excessive positive droplets are not considered suitable for absolute quantification by digital PCR. The Ct values for the two targets in qPCR, calculated copy numbers in each ddPCR reaction, and percent positive droplets and the number of positive droplets are shown. Both assays were done in duplicate, and average values are shown.

Number	Sample No.	COX Ct	LAS Ct	# Copies	Percent droplets	Positive	Positive droplets
		qPCR		ddPCR			
1	884	17.54	24.79	16540	50		8023
2	1548	16.21	24.51	16360	50		7597
3	1595	16.90	25.03	12880	42		6130
4	469	16.27	26.28	11960	40		5738
5	2103	17.00	25.31	11120	38		5206
6	2115	16.36	25.53	11100	38		4003
7	1143	16.68	25.24	10740	37		4925
8	1293	16.28	24.68	10620	36		5969
9	871	17.13	24.41	10580	36		5945
10	2263	16.41	25.25	10440	36		6038
11	2040	16.27	25.23	10260	35		5359
12	1670	16.92	24.24	9420	33		4973

13	1433	17.48	25.08	9220	32	4081
14	1765	17.11	26.12	9160	32	4871
15	706	17.19	24.39	8300	30	4112
16	1310	16.66	27.41	6820	25	3994
17	2493	15.97	26.76	6720	25	3133
18	462	17.19	25.13	5760	22	3240
19	2417	17.02	28.61	5560	21	2921
20	201	16.34	27.04	4860	19	3103
21	779	17.14	28.23	4700	18	3068
22	2278	16.71	26.78	3740	15	2241
23	2349	16.37	27.39	3580	14	1456
24	2113	16.13	27.65	3440	14	1872
25	556	16.32	28.42	1820	7	1124
26	345	16.97	29.43	1108	5	822
27	107	16.86	29.25	1016	4	592
28	1621	17.05	34.01	780	3	448
29	505	16.98	30.02	750	3	503
30	2066	16.53	30.16	374	2	221
31	714	16.99	29.35	332	1	226
32	2306	16.44	29.57	254	1	138

Table S3. Comparison of qPCR assays of ten-fold dilutions of a citrus DNA extraction positive for CLas. Dilutions were prepared using a healthy citrus DNA extraction (H) as the diluent. Two targets of CLas (16S and RNR) were assayed using either COX or MDH as reference genes. Average cycle threshold (Ct) values of three replications are shown. Numbers in parenthesis indicate standard deviation. In low-titer situations, Ct values for RNR (MDH as reference gene) were 2 Ct values lower than the assay detecting 16S with COX as reference (bolded and underlined data). Graphic representations are shown in Figures 3 and 4.

Dilution	16S & COX		16S & MDH		RNR & COX		RNR & MDH	
	COX	16S	MDH	16S	COX	RNR	MDH	RNR
10^{-0}	17.83 (0.06)	23.74 (0.14)	24.69 (0.11)	23.28 (0.08)	17.9 (0.06)	23.87 (0.04)	25.56 (0.57)	23.74 (0.05)
10^{-1}	19.25 (0.04)	28.14 (0.21)	26.56 (0.05)	26.48 (0.04)	19.4 (0.08)	27.7 (0.04)	26.93 (0.11)	26.82 (0.08)
10^{-2}	19.58 (0.03)	31.86 (0.15)	27.01 (0.05)	29.81 (0.1)	19.71 (0.03)	31.43 (0.02)	27.43 (0.13)	30.29 (0.19)
10^{-3}	19.3 (0.03)	36.01 (0.15)	26.94 (0.07)	34.38 (1.04)	19.53 (0.05)	34.69 (0.8)	27.31 (0.06)	33.7 (0.58)
10^{-5}	19.16 (0.02)	39.58 (0.58)	26.85 (0.09)	39.11 (1.55)	19.4 (0.01)	38.82 (1.77)	27.15 (0.06)	37.58 (0)
10^{-6}	19.6 (0.04)	0 (0)	27.17 (0.02)	0 (0)	19.7 (0.12)	0 (0.89)	27.55 (0.06)	0 (0)
10^{-7}	19.13 (0.07)	0 (0)	26.69 (0.02)	0 (0)	19.31 (0.09)	0 (0)	27.09 (0.06)	0 (0)
H	18.88 (0.03)	39.94 (0.11)	26.39 (0.09)	0 (0)	19.07 (0.1)	0 (0)	27.05 (2.32)	0 (0)
